# Evaluation of a Community-Based Trapping Program to Collect *Simulium ochraceum sensu lato* for Verification of Onchocerciasis Elimination

**DOI:** 10.1371/journal.pntd.0003249

**Published:** 2014-10-23

**Authors:** Mario A. Rodríguez-Pérez, Monsuru A. Adeleke, Isabel C. Rodríguez-Luna, Eddie W. Cupp, Thomas R. Unnasch

**Affiliations:** 1 Centro de Biotecnología Genómica, Instituto Politécnico Nacional, Reynosa, Tamaulipas, México; 2 Facultad de Medicina, Universidad Autónoma de Nuevo León, Monterrey, Nuevo León, México; 3 Public Health Entomology and Parasitology Unit, Department of Biological Sciences, Osun State University, Osogbo, Nigeria; 4 Department of Entomology and Plant Pathology, Auburn University, Auburn, Alabama, United States of America; 5 Global Health Infectious Disease Research Program, Department of Global Health, University of South Florida, Tampa, Florida, United States of America; Technical University of Mombasa, Kenya

## Abstract

**Background:**

Collection of the black fly vectors of onchocerciasis worldwide relies upon human landing collections. Recent studies have suggested that the Esperanza Window Trap baited with a human scent lure and CO_2_ had the potential to replace human hosts for the collection of *Simulium ochraceum sensu lato* in Southern Chiapas focus, Mexico. The feasibility of utilizing these traps in a community-based approach for the collection of *S. ochraceum s.l.* was evaluated.

**Methodology/Principal findings:**

Local residents of a formerly endemic extra-sentinel community for onchocerciasis were trained to carry out collections using the traps. The residents operated the traps over a 60-day period and conducted parallel landing collections, resulting in a total of 28,397 vector black flies collected. None of the flies collected were found to contain parasite DNA when tested by a polymerase chain reaction assay targeting a parasite specific sequence, resulting in a point estimate of infection in the vectors of zero, with an upper bound of the 95% confidence interval 0.13 per 2,000. This meets the accepted criterion for demonstrating an interruption of parasite transmission.

**Conclusions/Significance:**

These data demonstrate that Esperanza Window Traps may be effectively operated by minimally trained residents of formerly endemic communities, resulting in the collection of sufficient numbers of flies to verify transmission interruption of onchocerciasis. The traps represent a viable alternative to using humans as hosts for the collection of vector flies as part of the verification of onchocerciasis elimination.

## Author Summary

Onchocerciasis, or river blindness, is a neglected tropical disease that has been identified by the international community as a candidate for elimination. Both the criteria for verification of elimination and for post-treatment surveillance developed by the international community rely heavily on the use of entomological metrics. Large numbers of vector black flies must be collected to satisfy these metrics. The current standard method for collection of vector black flies for this purpose is human landing collections, is both inefficient and potentially hazardous to the collectors. Here, we report studies evaluating a community-based trial of an inexpensive trap made largely from locally available materials for the replacement of fly collection teams. Traps were provided to residents of a formerly onchocerciasis endemic community in Mexico, and the residents allowed to operate the traps over a 60 day period. The number of flies collected was sufficient to meet the current international criteria necessary to verify that the community was free of *O. volvulus* transmission. These findings suggest that community based operation of this simple trap might replace human landing collections in the process of verifying the interruption of transmission of onchocerciasis.

## Introduction

Onchocerciasis or river blindness is a disease that results from infection with the filarial parasite *Onchocerca volvulus*. *O. volvulus* is a vector-borne infection parasite transmitted by blackflies of the genus *Simulium*. The disease has historically been a very serious problem in the developing world, where it is has had a devastating socio-economic in the most afflicted communities, primarily in sub-Saharan Africa and to a lesser extent in the 13 foci of Latin America [1]–[5]. The programs attempting to eliminate onchocerciasis all currently rely primarily on community-wide treatment of the endemic populations with ivermectin (Mectizan donated by Merck & Co.). Quarterly, semi-annual and annual regimens of Mectizan treatment have been successful in interrupting, and in some cases eliminating transmission of the parasite in different situations [6]–[12].

The elimination guidelines developed by the Onchocerciasis Elimination Program for the Americas (OEPA) and the World Health Organization (WHO) rely to a large extent on measuring the prevalence of the infective stage of *O. volvulus* larvae (L3) in the vector populations to determine if transmission has been interrupted [13], [14]. In Latin America, the current guidelines state that to in order to declare that transmission in interrupted, the prevalence of L3 in the vector population must be low enough so that the upper bound of the 95% confidence interval (95% CI) of the proportion of flies carrying L3 is less than 1/2,000 per endemic community [14]. To meet this criterion, at least 6,000 flies have to be tested from each endemic community [15], [16]. The primary method for collecting host-seeking black flies has been human landing collections [17]–[19]. This requires stationing adult volunteer collectors in areas of high *Simulium* densities and collecting black flies that attempt to land and blood-feed upon the collector. Apart from the fact that this procedure has been criticized because of the potential risk of infection for the collectors [20], it can be difficult for the human landing collectors to capture the large number of flies needed to demonstrate that transmission has been interrupted. Thus, replacing human landing collection is ever more important as the focus of the onchocerciasis community shifts from control to elimination, and in some cases post-treatment surveillance [21].

In a recent study, a novel trap design (the Esperanza Window Trap) collected numbers of black flies similar to those obtained by a team of human landing collectors [22]. Here, we evaluated the Esperanza Window Trap using a community-based implementation plan. Furthermore, we report the results of a PCR pool-screening assay for *S. ochraceum* s.l. collected using both Esperanza Window Traps and human landing collections. The data suggest that *O. volvulus* transmission has been interrupted in this extra-sentinel community of the former Southern Chiapas focus in México.

## Materials and Methods

### Study Site

Studies were carried out in the village of Las Golondrinas, Chiapas, Mexico (15°25′59″N; 92°39′06″W; elevation 890 m). Las Golondrinas is in Southern Chiapas, which historically was the largest of the three foci of onchocerciasis in Mexico. Transmission of *O. volvulus* was widespread in this area prior to its elimination as a result of intensive mass treatment with Mectizan [23]–[25]. The studies were conducted during the 2013 dry season, when high populations of parous *Simulium ochraceum sensu lato*, the primary vector of *O. volvulus* in the region occur [26].

### Trap Design

**Figure 1:**
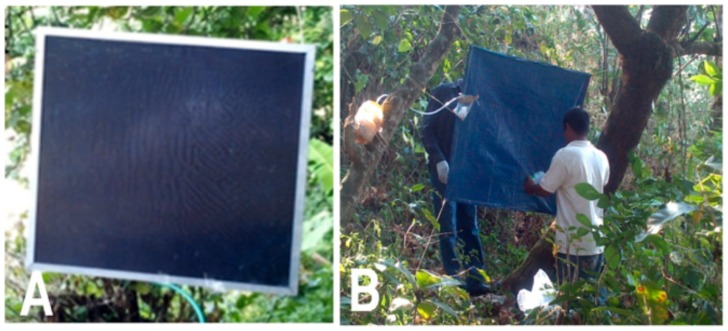
Original and modified Esperanza trap designs. Panel A: Original acrylic sandwich design. Panel B: Modified tarpaulin design.

Previous studies evaluating different trap designs for the collection of *S. ochraceum s.l.* demonstrated that the Esperanza Window Trap baited with a commercial mosquito lure (BG-Lure; Biogents AG, Regensburg, Germany) and CO_2_ was an effective design for the collection of these black flies [22]. The original design of the Esperanza Window Trap consisted of blue satin fabric sandwiched between two sheets (2 mm thickness) of clear acrylic, supported by an aluminum frame. This design was found to be relatively expensive to construct (roughly $50 USD per trap), heavy, and difficult to manipulate in the field. As a first step in utilizing the Esperanza Window Trap in a large-scale field trail, the design was altered. The modified design consisted of a 1.0×1.0 m piece of blue plastic tarpaulin coated with Tangle Trap glue and mounted upon a window screen frame, baited with the BG-Lure and organically-generated CO_2_ (Figure 1).

### Trap Evaluations

The original and tarpaulin designs were baited with BG-Lure attractant and organically generated CO_2_ as previously described [22]. The traps were deployed between 7 AM and 12 PM for three days, and their positions rotated every day. Collections were retrieved once daily (12 PM). Flies were removed from the traps by dissolving the Tangle Trap glue with odorless mineral spirits. Flies were stored in the solvent for subsequent morphological identification and PCR pool screening. To determine if the collections of the two trap designs were significantly different, a one-way Analysis of Deviance was carried out using the SAS GENMOD procedure with distribution type set to the Negative Binomial, which is the standard model for over-dispersed Poisson data [27]. None of the goodness of fit statistics indicated any lack of fit.

Based upon the results obtained from the initial evaluations of the trap designs, a large-scale study was conducted evaluating the effectiveness of the traps when operated by community members. This study employed residents of the communities to operate and maintain the traps with minimal supervision. Volunteers from the community were identified and provided instruction on the operation of the traps and identification and removal of *S. ochraceum* s.l. from the trap surfaces. The teams were also instructed how to conduct human landing collections. Collections were carried out over a 60-day period within the village itself and in a nearby coffee plantation. A small intermittent stream flowed through each collection location, which served as a breeding site for *S. ochraceum* s.l. [22]. Two teams were employed to collect flies and conduct human landing collections at each site. Each team consisted of two local residents.

**Figure 2:**
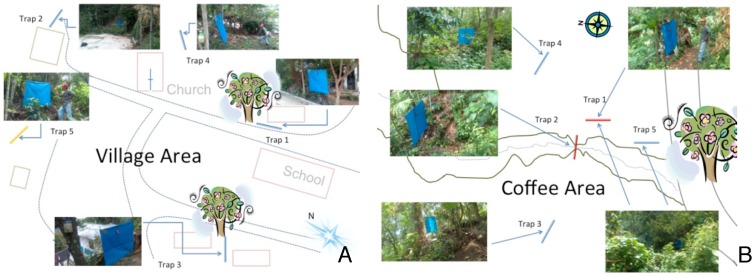
Position of traps at the two collection locations. Panel A: Trap positions in the village. Panel B: Trap positions in the coffee plantation. Arrows indicate the trap position relative to local landscape features and photos show the actual trap placement.

Five Esperanza window traps were set out in each location (the village and coffee plantation) in a circular pattern with the stream at the center, (Figure 2, Panels A and B). The traps were placed roughly 2–7 m from the breeding site, separated by a distance of 10–20 m from one another. The traps were run from 8 AM until 12 PM daily. Before every trapping session, any black flies that were collected during the non-experimental periods were removed from the traps. Collections were retrieved once daily (12 PM). While the traps were being run, the team at each plot conducted human landing collections, as previously described [7]. Human landing collections were carried out approximately 5 m from the traps. This distance was chosen to minimize the interference among traps and human collectors while still drawing from the same host-seeking black fly population. The team rotated the positions where the human landing collections were carried out among the positions where the traps were placed, to control for location-specific variation in the number of flies present among the trap sites.

### Ethics Statement

The human landing collection procedures were reviewed and approved by the appropriate institutional review boards. These included the Bioethics Committees of the Center for Research and Development in Health Sciences of the Autonomous University of Nuevo León (Monterrey, Nuevo León, Mexico), and the Institutional Review Board of the University of South Florida. Written informed consent was obtained from all participants.

### PCR Pool-Screening Assay

Flies were grouped into pools containing a maximum of 200 individuals per pool and the heads and bodies separated as previously described [28]. The separated bodies were using a PCR assay specific for *O. volvulus*, as previously described [29]. Screenings focused on pools of bodies, as previous studies have shown that infection rates in bodies, which contain multiple life cycle stages of the parasite, provide a more sensitive indicator of parasite-vector contact than testing heads, which only contain L3 larvae [28], [30]. As no body pools were found to be positive, head pools were not screened.

### Data Analysis

PoolScreen v2.0 [31] was used to estimate the upper bound of the 95% confidence interval for the prevalence of flies carrying *O. volvulus*. The landing rate measured from the human collections was used to estimate the biting rate. This probably overestimated the biting rate, as some of the flies land but do not successfully take a blood meal. Thus, the biting rate calculations provided below over-estimate the biting rate to some extent. The seasonal transmission potential [STP] was calculated as the product of the seasonal biting rate, the proportion of flies carrying L3 larvae in the transmission late dry season (from April through May), and the average number of L3 larvae in each infective fly. As previously discussed, after multiple rounds of Mectizan treatment, the number of infective larvae present in each infective fly was assumed to be one [7], [8], [25]. Because *S. ochraceum s.l.* females were not collected throughout the year, it was not possible to precisely calculate the annual transmission potential (ATP). However, given that the infected black flies are uncommon outside of the late dry season in Latin America [19], [26], the STP probably provided a fairly accurate estimate of the ATP.

The GLIMMIX procedure in the SAS program package (SAS version 9.4 13w18 Media), was used to adjust the number of flies caught to a negative binomial distribution, and the least square means of the fly collections in the village and coffee plantation were calculated and compared. Using the same procedure, paired least square means of the collections from each trap position were calculated and compared using t-tests. GLIMMIX was also used to test for any interaction between the collection rates, as measured by the human landing collections and the trap positions.

## Results

**Figure 3:**
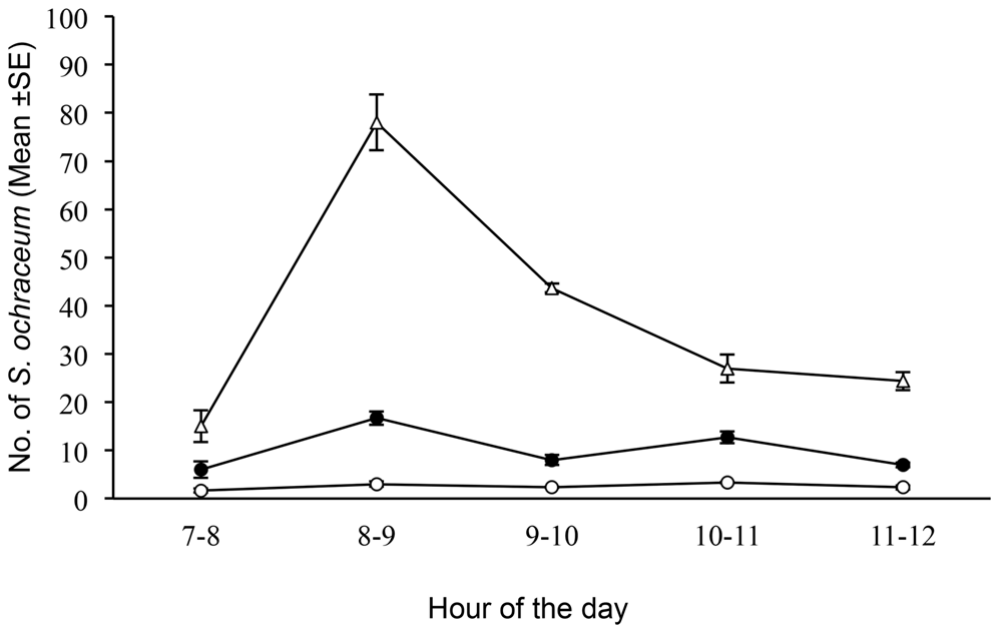
Performance of the original and tarpaulin based trap designs. Symbols present the mean and error bars of the standard error of collections taken over a three-day period. Open circles = collections by the original acrylic sandwich design. Solid circles = collections by the modified tarpaulin based design. Open triangles = human landing collections.

As a first step in adapting the Esperanza Window Trap for use in a community-based trial, a simplified version of the trap was constructed. This simplified design, consisting of a piece of blue plastic tarpaulin coated with Tangle-Trap insect paste and baited with BG-Lure and organically-generated CO_2_ (Figure 1) was evaluated in side-by-side comparisons with the original design, as described in Materials and Methods. The simplified design collected roughly five times as many flies as the original trap design (Figure 3; p<0.001, Tukey-Kramer adjusted), indicating that it was at least as effective as the original.

The blue tarpaulin traps were then evaluated in a community-based format, as described in Materials and Methods. Two teams consisting of two local individuals each were trained in the operation of the traps and in conducting human landing collections. One team was given responsibility for maintaining five traps and carrying out human landing collections at a site located in the village proper, while the second team maintained five traps and carried out human landing collections in a site located in a nearby coffee plantation (Figure 2). A total of 9,986 flies were collected using the traps at both sites (Table 1).

**Table 1:** Mean ± standard error (SE) of the daily number of *Simulium ochraceum s.l.* captured over 60 days.

Location	Trap collection (total from 5 traps per location)	Mean collection ± SE^1^	Human landing collections	Mean collection ± SE^2^
Village	5,941	18.14±0.40	11,200	184.00±11.20
Plantation	4,045	12.60±0.30	9,797	162.25±9.88
Total	9,986	16.64±0.42	20,997	174.97±8.40

1 Flies/trap/day.

2 Flies/team/day.

**Figure 4:**
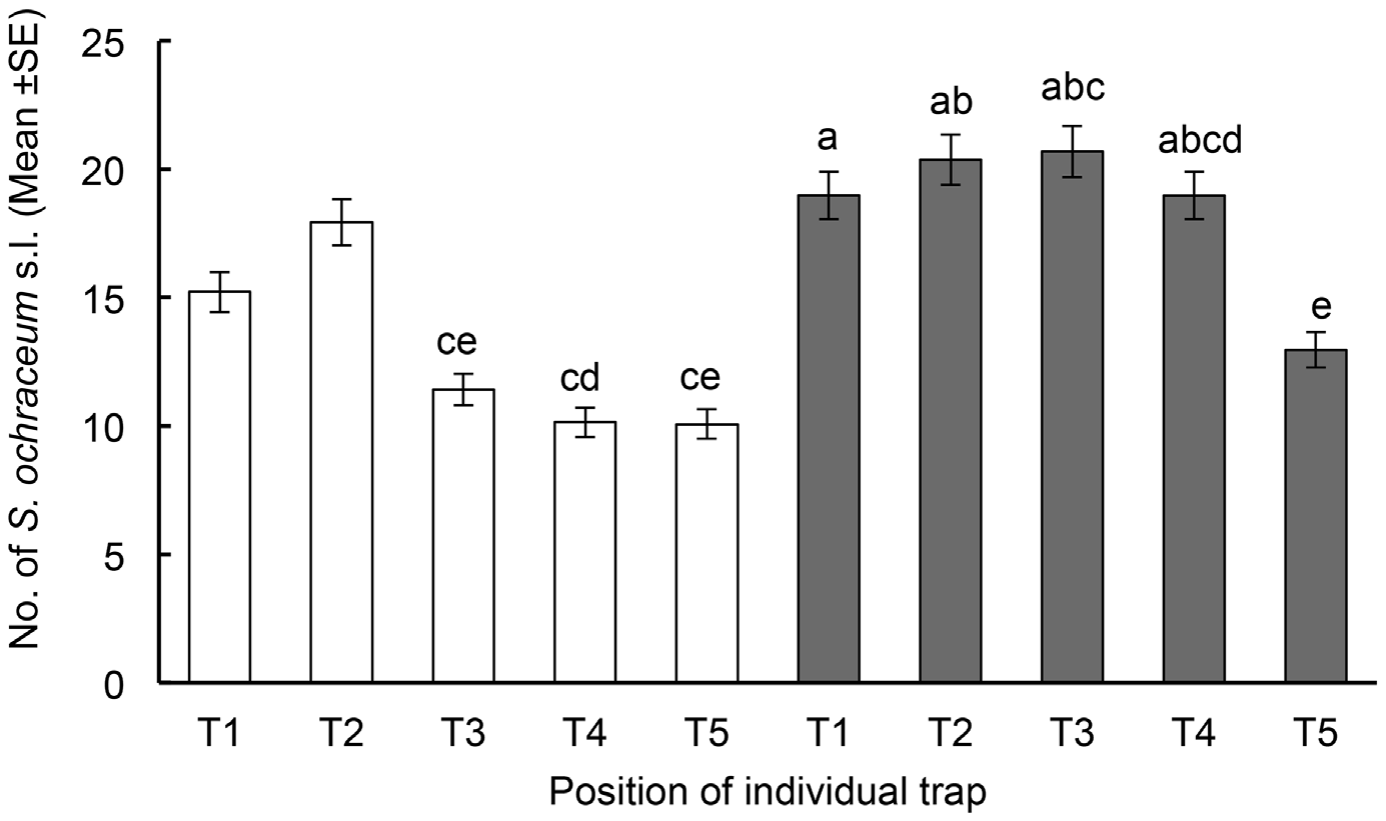
Mean (± Standard Error) of the number of flies caught by the five individual traps in coffee plantation (bars in white) and village (bars in grey). Collections among the traps at each site were compared in a pairwise fashion and the statistical significance of differences between the paired trap collections assessed using a t-test. Means with the same letter were not significantly different (p>0.05).

The collection data (fly counts per trap) were found to conform to a negative binomial distribution, and were thus examined using a generalized linear mixed model. The model:
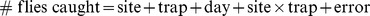
was found to fit the data well (−2 log likelihood chi square value of 3826.16) with no evidence for over-dispersion (chi-square coefficient of Pearson/degrees of freedom = 0.98, when a coefficient of >1.0 indicates over-dispersion). The mean number of *S. ochraceum* s.l./trap day was significantly higher for traps located in the village than for the traps located in the coffee plantation (18.14±0.40 versus 12.60±0.30, respectively; p<0.0001). Significant differences were also noted among the number of flies collected from the traps at each location (Figure 4). For example, the traps at positions T1 and T2 in the coffee plantation collected significantly more flies than the traps places at positions T3–T5. Conversely, the trap placed at position T5 in the village collected significantly fewer flies that the other four village locations (Figure 4).

The human landing teams collected significantly larger numbers of flies at both sites than did the five traps combined at each site (Table 1; p<0.001). Similar to the trap collections, human landing collections in the village were higher than in the plantation, though this difference did not reach statistical significance (F-value = 2.16, p = 0.05). No evidence for any interaction between the human landing collections and the trap collections at a given site were seen (F = 2.14, p>0.05).

A total of 7,400 flies collected by the traps were tested by pool screen PCR for the presence of *O. volvulus*, a number that was sufficient to comply with the OEPA guideline of having at least 6,000 flies tested from each community. In addition, 20,997 flies collected by the human landing collections were tested. All body pools were negative for *O. volvulus* DNA, which suggested that no parasite-vector contact was occurring, and the point prevalence of flies carrying *O. volvulus* was zero (Table 2). The upper bound of the 95% confidence interval of the infection rate was 0.13/2000, well below the threshold of 1/2,000 mandated by the OEPA guidelines. The upper bound of the 95% CI for the STP was calculated to be 0.96 L3s/person/season.

**Table 2:** Results of PCR pool screening for collected flies.

Method	No. of flies	No. of pools^1^	No. positive pools	Prevalence of infected flies^2^
Esperanza Window traps	7,400	37	0	0 (0.51/2,000)
Human landing collections	20,997	105	0	0 (0.18/2,000)
Total	28,397	142	0	0 (0.13/2,000)

1 Maximum of 200 flies per pool.

2 Upper number = point estimate and lower number = upper bound of the 95% CI of the prevalence of infected flies.

## Discussion

In previous studies, we reported the development of a trap platform, designated the Esperanza Window Trap, which appeared to have potential as a replacement for human landing collections [22]. However, the bulky and costly design of this trap made it difficult to deploy in the field. The studies reported above suggest that a simplified design, consisting of a piece of blue plastic tarpaulin, was at least as effective as the original design. The tarpaulin design of the Esperanza Window Trap is lightweight and inexpensive (roughly $5.00 USD per trap). Furthermore, it is constructed of materials that are readily available in developing countries and is highly portable. This trap design therefore represents a very attractive alternative to human landing collections for the collection of the black fly vectors of *O. volvulus*.

Given that these traps are intended to be used as a component of the surveillance activities to verify the interruption of *O. volvulus* transmission and to monitor for recrudescence of transmission in the post-endemic era, they will have to be operated routinely in the endemic communities. Employing residents of the communities themselves to operate the traps is logistically practical, since such a community based approach would be less expensive than devising a vertically integrated program involving small teams of highly trained individuals to operate the traps. In addition, employing local residents would permit a larger distribution of trap activities and enhance community involvement in the onchocerciasis elimination agenda. The data presented above suggest that such a community-based strategy may be feasible. Overall, the five traps set at each site combined to collect approximately 50% as many flies as did the human landing collectors. However, the performance of the traps, when operated by the residents, was approximately 20% of the catch when compared to when the traps were operated by trained entomologists [22]. Despite this difference, the traps, when operated by the residents, still represented a viable alternative to human landing collections. This is because a single person can easily maintain five traps, and a two-man human landing collection team can therefore maintain 10 traps per day. Furthermore, maintaining the traps only requires visits in the morning and evening, freeing up the rest of the day for other activities by the individuals maintaining the traps. Thus, when maintained by local residents, the traps can be operated throughout the transmission season, resulting in better estimates of fly activity than can be obtained by human landing teams, which generally cannot operate continuously in every community through the transmission season.

The performance of the individual traps differed significantly among the locations at both the village and plantation sites. This is perhaps not surprising, as vision plays an important role in black fly host seeking behavior, and visibility varied from location to location due to the density of the local vegetation. Although the traps in the village were in more open areas, and thus more susceptible to changing climatic conditions such as sun, rain, and wind, it appeared that traps placed in the village were in general more effective than traps placed in the coffee plantation where human activity was lower than in the village. The data suggest that trap placement will play an important role in catch efficiency and that traps placed in more open areas with greater human activity may perform better than those placed in areas with dense vegetation.

In Las Golondrinas, pre-control entomological data were available and Mectizan treatment has reduced transmission by greater than 99% when compared to the levels that existed prior to the start of the elimination program (about 20 L3s per person per year) [23], [24], [28]. This meets the criterion developed by WHO indicating a “near absence” of transmission for areas where such pre-treatment data exist.

OEPA recommends the use of ATP to assess the status of onchocerciasis transmission, because ATP takes into account both the biting rate and the prevalence of infective flies. Estimates of the ATP necessary to maintain the parasite population (the transmission breakpoint) range from 5 to 54 L3/person/year based on mathematical modeling studies [32] and from 7.6 to 18 L3/person/year based on field observations [13], [33]. The point estimate of STP in Las Golondrinas was zero, and the upper bound of the 95% confidence interval for the STP was significantly below all of these estimated transmission breakpoints. It should be noted that the STP was calculated only on collections carried out during the peak of the transmission season, and therefore may underestimate the actual ATP to some extent. However, previous studies of the transmission of *O. volvulus* in Latin America have suggested that transmission outside of the peak season is zero or near zero [19]. Thus, the upper bound of the 95% CI of the STP reported here probably represents a fairly accurate, though somewhat low estimate of the upper bound of the ATP.

In conclusion, the data suggest that transmission remains undetectable in Las Golondrinas, a village located within an area where transmission of *O. volvulus* was historically quite high [25]. The entomological findings from this village continue to meet the criteria developed by the international community for suppression of transmission [13]–[15] two years after treatment was suspended. If permanent interruption of transmission in the other sentinel and extra-sentinel communities of the Southern Chiapas focus can be confirmed during the post-treatment surveillance evaluations to be completed in 2014, it will culminate in the verification of the complete elimination of this scourge from Mexico.
